# Effect of Exoskeleton-Assisted Rehabilitation Over Prefrontal Cortex in Multiple Sclerosis Patients: A Neuroimaging Pilot Study

**DOI:** 10.1007/s10548-021-00858-w

**Published:** 2021-06-28

**Authors:** V. Sulpizio, M. Berchicci, F. Di Russo, G. Galati, M. G. Grasso, M. Iosa, G. Lucci, S. Paolucci, M. Ripani, Sabrina Pitzalis

**Affiliations:** 1 Department of Cognitive and Motor Rehabilitation and Neuroimaging, Santa Lucia Foundation (IRCCS Fondazione Santa Lucia), Rome, Italy; 2 Department of Psychology, Sapienza” University of Rome, Via dei Marsi, 78, 00185 Rome, Italy; 3 Department of Movement, Human and Health Sciences, University of Rome “Foro Italico”, Rome, Italy; 4 Department of Human Sciences, Marconi University, Rome, Italy

**Keywords:** Human Body Posturizer, Multiple sclerosis, Functional magnetic resonance, Inferior frontal gyrus

## Abstract

Application of a passive and fully articulated exoskeleton, called Human Body Posturizer (HBP), has been demonstrated to improve mobility, response accuracy and ambulation in multiple sclerosis (MS) patients. By using functional magnetic imaging (fMRI) during a visuomotor discrimination task, we performed a pilot study to evaluate the effect of HBP over the neural correlates of motor and cognitive functions which are typically impaired in MS patients. Specifically, we tested the effect of a 6-week multidisciplinary rehabilitation intervention on two groups of MS patients: a control group who followed a standard physiotherapeutic rehabilitation protocol, and an experimental group who used the HBP during physical exercises in addition to the standard protocol. We found that, after treatment, the experimental group exhibited a significant lower activity (as compared to the control group) in the inferior frontal gyrus. This post-treatment activity reduction can be explained as a retour to a normal range, being the amount of iFg activity observed in the experimental patients very similar to that observed in healthy subjects. These findings indicate that the use of HBP during rehabilitation intervention normalizes the prefrontal activity, mitigating the cortical hyperactivity associated to MS.

## Introduction

Multiple sclerosis (MS) is a progressive, inflammatory and neurodegenerative disease that causes demyelinating lesions, axonal degeneration and formation of sclerotic plaques. It is caused by a complex interplay between genetic and environmental factors and it is characterized by unpredictable course of disease (see Trapp and Nave 2008 for a review). Patients may experience a wide range of symptoms such as dizziness, fatigue, loss of energy, feeling of exhaustion, decrease in motivation, mood disorders, spasticity, gate and balance difficulties, visual, bladder and bowel problems, sexual dysfunction, pain, tremor (Samkoff and Goodman 2011). Other common deficits in SM are dysarthria, dysphagia and cognitive disorders, such as memory, attention and executive dysfunctions (Merson and Rolnick 1998).

Beyond pharmacological therapies, several studies have investigated different therapies for treating MS symptoms, including passive strategies such as heat and/or cold therapy, supportive braces, and active strategies such as exercise, biofeedback relaxation, and psychosocial interventions (Khan 2007; Khan 2013). A more recent review highlighted the importance of structured multidisciplinary rehabilitation programs and physical therapy to improve functional outcomes and quality of life of MS patients (Amatya et al. 2019).

Altered balance/stability during walking is common in people with multiple sclerosis. Up to 50% of MS patients require walking aids and 10% are wheelchair-bound 15 years following the initial diagnosis (Al-Omaishi et al. 1999). Assisted rehabilitation training technologies, such as body weight support and robot-driven orthoses, may provide further improvements in MS patients’ neurorehabilitation, by allowing continuous stabilization of balance and a more accurate control and support of walking movements.

**Fig. 1:**
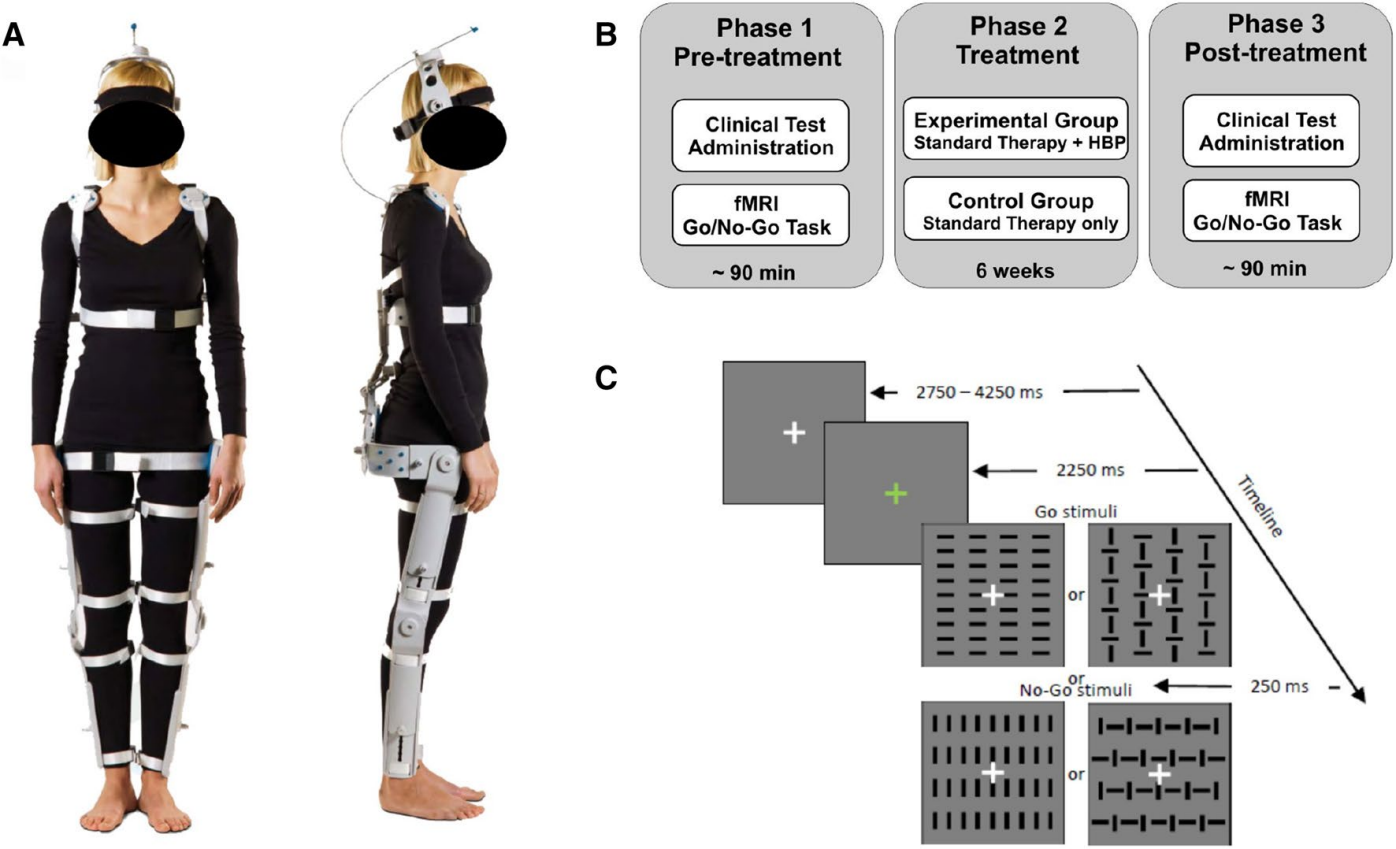
**A** Human Body Posturizer (HBP). Frontal and lateral views of the HBP exoskeleton worn by an actor. **B** Flow-chart of the experimental protocol. **C** Schematic illustration of the stimulus sequence of a go/no-go trial; in every trial only one of the four depicted stimuli at time is displayed

Among these technological devices, we recently tested the beneficial effects of the ‘‘Human Body Posturizer’’ (HBP) system (Fig. 1A), which is a passive and fully articulated exoskeleton able to improve the walking performance in both healthy and clinical population. Indeed, previous studies showed its beneficial effects on postural dynamics also in healthy subjects (Colaiacomo et al. 2011; Ciccarelli et al. 2012). These studies revealed that the HBP may increase the degree of symmetry in trunk and lumbar regions of the spinal column and reduce the risk of falling in the elderly.

Given these promising results on healthy people, we were encouraged to test the HBP also on MS patients. In a first event-related potential (ERP) study by Di Russo et al. (2013) we observed that one single application of the HBP was able to improve mobility, ambulation and response accuracy in MS patients. Importantly, these beneficial effects were associated to changes in brain activity, especially in the prefrontal cortex, as revealed by electrophysiological measures. As hypothesized by Di Russo et al. (2013), the HBP would act on proprioceptive receptors so that signals on the correct posture are transmitted to supra-axial nerve centers, to be then integrated and interpreted in the central nervous system.

We further confirmed the beneficial effect of HBP on MS patients in another recent ERP study (Berchicci et al. 2019). This study showed that the HBP intervention is more effective than the standard rehabilitation protocol, especially reducing disability status and fatigue, likely stimulating the brain centers underpinning cognitive processing, as revealed by EEG changes in the prefrontal cortex.

Here, we used the same pilot randomized controlled trial as in Berchicci et al. (2019) to evaluate the use of the HBP to assist the rehabilitation process in patients with MS by testing, for the first time to our knowledge, the effect of this treatment on the brain activity as measured by functional magnetic resonance imaging (fMRI). fMRI has becoming a powerful tool to investigate brain function changes following rehabilitation program. For example, many studies of cognitive rehabilitation efficacy in MS have recently applied fMRI to establish outcome (see Chiaravalloti et al. 2015 for a review). As in Berchicci et al. (2019), we tested the effect of a 6-week multidisciplinary rehabilitation intervention on two groups of MS patients: a control group who followed a standard rehabilitation protocol, and an experimental group who used the HBP during physical exercises in addition to the standard protocol. We assessed the effect of these treatments observing, with high anatomical definition, fMRI correlates of functional changes within the prefrontal cortex. To compare and extent previous ERP studies, we used the same discriminative visuo-motor task (Go/No-go task) used in Di Russo et al. (2013) and Berchicci et al. (2019). This task is frequently used to assess cognitive processes, such as proactive and reactive inhibition, decision-making, motor preparation, speed processing and behavioral execution, most of them controlled by the prefrontal cortex (Aron et al. 2011). These functions have been shown to be the cognitive capacities that are impaired in MS (Benedict et al. 2006; Rao et al. 1991), although the most common cognitive deficits in MS include mental processing speed, episodic memory and learning new information (Chiaravalloti and DeLuca 2008; Benedict and Zivadinov 2011).

Here we predict that the rehabilitation reinforced by the HBP could ameliorate mobility and prefrontal executive functions, which are impaired in MS patients. Since we previously observed that the use of HBP in MS patients induces changes in the prefrontal areas only using electro-cortical measures that have low spatial resolution (Di Russo et al. 2013; Berchicci et al. 2019), here we expect to find, with the high resolution of neuroimaging methods, measurable and anatomically precise changes in the post-treatment brain activity of the prefrontal cortex. We also predict that this effect on the cortical activity might drive cognitive and physical improvement during rehabilitation training.

## Methods

### Participants

As a pilot study we tested a small group of MS patients, which were the same as those recruited in a previous electroencephalographic ERP study (Berchicci et al. 2019). All these patients were diagnosed according to the revised McDonald criteria (Polman et al. 2011). A total of nine patients participated to the fMRI study; they were randomly assigned to the control or to the experimental group. The two groups were age- and gender-matched (univariate tests showed lack of significant differences p > 0.8), as follows: control group: N = 4, 2 females, mean age 50.3 ± 7.3 years; experimental group: N = 5, 2 females, mean age 49.0 ± 7.3 years. The MS patients were selected based on the absence of other neurological disorders and gross visual pathologies, and with Expanded Disability Status Scale (EDSS) between 5 and 7. Data obtained in both control and experimental groups were qualitatively compared to that obtained in young healthy controls (16 volunteers, eight females, mean age 26.0 years, SD = 4.4) enrolled in our previous study in which the same task was used (Di Russo et al. 2016). All participants were right-handed (Edinburgh handedness inventory, Oldfield 1971). The participants’ written consent was obtained according to the Declaration of Helsinki after approval by the ethical committee of the IRCCS Santa Lucia Foundation.

### Experimental Procedure

The experimental procedure, which is schematically described in Fig. 1B, includes an intervention session, and two identical test sessions, i.e., pre- and post-treatment.

#### Intervention

The intervention protocol was described in detail in Berchicci et al. (2019). Briefly, all patients underwent a 6-week multidisciplinary rehabilitation training, consisting of in twice-daily physiotherapy treatments, each lasting 45 min, performed 5 days a week. The intervention consisted of muscle stretching, postural alignment especially at the height of axial segments, active-assisted mobilizations and neuromuscular facilitations to improve motor recruitment. Balance training was performed in different positions during standing and dynamic tasks using exercises with a progressive restriction of the support base and the use of unstable surfaces like wobble boards, balance pads or stability balls. These exercises were carried out with both eyes-closed and eyes-open. The control group executed this training wearing normal clothes, while the experimental group wore the HBP (see Fig. 1A). The HBP was calibrated for each patient regulating the subclavian and lumbosacral thrusts in order to improve the rachis straightening reactions and the stability between shoulder and pelvic girdles. Patients were instructed to perceive the proprioceptive adjustments and stimulations offered by the exoskeleton. The training program was devised and coordinated by the treating physicians and administered by qualified physiotherapists from Santa Lucia Foundation.

#### Clinical Assessment

Before and after treatment, all MS patients were administered a battery of tests.

The performance-based tests included: (1) 2 min Walk Test (2-WT), a measurement of endurance by assessing walking distance over 2 min (Gijbels et al. 2011); (2) Timed 25 Foot Walk Test (T25-FW), a quantitative mobility and leg function performance test based on walking timing for a distance of 25 feet (Motl et al. 2017); (3) Tinetti test, a measure assessing gait and balance ability (Tinetti et al. 1986); (4) Berg Balance Scale (BBS), a balance test assessing the performance of a functional task (Berg et al. 1989).

The functional tests included: (1) Barthel scale, a measurement of activities of daily living (Mahoney and Barthel, 1965) and (2) Rivermead Mobility Index (RMI), which is a mobility test (Collen et al. 1991).

The neurological test consisted in the Expanded Disability Status Scale (EDSS), which quantifies disability in eight functional systems (i.e. pyramidal, cerebellar, brainstem, sensory, bowel and bladder, visual, cerebral, other) (Kurtzke 1983).

Moreover, all MS patients were also administered a Fatigue Severity Scale (FSS), a self-report questionnaire designed to measures the severity of fatigue and its effect on a person's activities and lifestyle (Krupp et al. 1989).

Table 1 summarizes the relevant clinical data obtained before and after treatment for each group of patients. Statistical difference as a function of session (pre vs. post) independently of group was observed for 2-WT, Tinetti, BBS and Barthel tests. A more specific effect of HBP treatment was observed on FSS scores, indicating a significant fatigue reduction from pre- to post-treatment for the experimental group only. Further details about performance-oriented, functional and neurological data are available in Berchicci et al. (2019).

**Table 1:** Clinical assessment. Mean scores $\pm$ SD were provided for performed-based, functional and neurological tests as a function of group and session

Measure	Pre test		Post test	
	Experimental group	Control group	Experimental group	Control group
2-WT*	57.4 ± 16.4	69.7 ± 20.5	66.0 ± 14.6	54.7 ± 12.4
T25-FW	15.5 ± 4.6	14.1 ± 7.6	12.1 ± 3.8	16.3 ± 6.1
Tinetti*	15.4 ± 4.6	24.3 ± 4.2	23 8 ± 4.9	22.5 ± 4.3
BBS*	37.0 ± 7.5	44.3 ± 7.6	44.6 ± 6.6	39.3 ± 7.3
Barthel*	73.2 ± 16.2	91.0 ± 5.7	88.4 ± 6.1	84.5 ± 4.6
RMI*	6.2 ± 1.3	9.3 ± 1.8	7.8 ± 1.3	7.8 ± 1.9
EDSS	6.3 ± 0.5	6.1 ± 0.4	6.2 ± 0.5	6.4 ± 0.4
FSS**	4.2 ± 0.2	6.1 ± 0.3	3.6 ± 0.5	6.2 ± 0.6

*Significant effect of session (pre vs. post), independently of group

**Interaction between session and group: pre < post only in the experimental group. More details in Berchicci et al. (2019)

#### Go-No Go Task

During both pre- and post-treatment fMRI sessions, participants performed a discriminative response task or DRT (Fig. 1C) that we have already used in previous ERP/fMRI coregistration studies to assess spatiotemporal dynamics of visuomotor control in healthy subjects (Di Russo et al. 2016; Sulpizio et al. 2017; Berchicci et al. 2020). Participants laid on their back in the scanner and with their right-hand positioned palm down on a push button board. Each acquisition scan started with the fixation cross (0.15° × 0.15° of visual angle) in the center of the screen, which never disappeared. Square patterns made by vertical and horizontal bars subtending 4° × 4° were presented for 250 ms on a dark grey background (Fig. 1C). The four patterns were displayed in a random sequence with equal probability (p = 0.25). Participants had to press a button with their right hand as fast and accurate as possible when a target appeared on the screen (go stimuli; p = 0.5) and withhold the response when a non-target appeared (no-go stimuli; p = 0.5).

Each trial started with a color change of the fixation cross, becoming either green or red and remaining for 2250 ms. If the fixation cross became green, after 2250 ms from the color changing, one of the four patterns was presented and remained on the screen for 250 ms. If the fixation cross became red, the participants were informed that after 2250 ms no pattern would be presented. This latter condition, also known as “relax”, was used as control condition for evaluating the cue-related orienting and perceptual brain activity. As a low-level baseline, we also included “null” trials, where the fixation-cross remained white for 2250 ms and no pattern was presented. The inter-trial interval (ITI) varied between 2750 and 4250 ms [mean 3500 ms, standard deviation (SD) 536 ms]. The order of presentation of go and no-go stimuli and trial types were randomized within each run. Each patient completed four functional acquisition scans, each including 18 go, and 18 no-go trials, as well as 18 relax and 8 null trials, for a duration of 6′12′.

### fMRI Apparatus and Procedures

Images were acquired using a 3 T Siemens Allegra MR system (Siemens Medical systems, Erlangen, Germany) operating at the Neuroimaging Laboratory, Foundation Santa Lucia, using a standard head coil. The participants lay on their back in the scanner, with their right-hand positioned palm down on a push button board to enable the index finger to move freely. Single-shot echo-planar imaging (EPI) images were collected using blood-oxygenation-level dependent imaging (Kwong et al. 1992) via a standard transmit receive birdcage head coil. Thirty contiguous MR axial slices were 4.5 mm thick (with a 0 mm gap, interleaved excitation order), with an in-plane resolution of 3 × 3 mm, oriented approximately in parallel to the AC–PC line. Sampling started from the superior convexity and included almost all of the cerebral cortex, excluding only the ventral portion of the cerebellum. In each scan, the first four volumes were discarded from the data analysis to achieve a steady state, and the experimental tasks were initiated at the beginning of the fifth volume. Each participant underwent six functional scans. The other imaging parameters were as follows: repetition time (TR) = 2 s; echo time (TE) = 30 ms, flip angle = 70°, 64 × 64 matrix, and bandwidth = 926 Hz/pixel. Structural images were collected using a sagittal magnetization-prepared rapid acquisition gradient echo (M-PRAGE) T1-weighted sequence: TR = 2 s, TE = 4.4 ms, flip angle = 8°, in-plane resolution = 0.5 × 0.5 mm, and slice thickness = 1 mm.

Stimuli were generated by a control computer located outside the MR room, running in-house software implemented in MATLAB (The MathWorks Inc., Natick, MA, USA). An LCD video projector with customized lens was used to project visual stimuli to a back-projection screen mounted inside the MR tube and visible through a mirror mounted inside the head coil. Presentation timing was controlled and triggered by the acquisition of fMRI images. Responses were given through push buttons connected to the control computer via optic fibers.

### Image Processing and fMRI Analysis

Images were preprocessed and analyzed using SPM12 (Wellcome Department of Cognitive Neurology, London, UK). Functional time series from each participant were first temporally corrected for slice timing using the middle slice acquired in time as a reference; the data were spatially corrected for head movements using a least-squares approach and six parameter rigid body spatial transformations. The data were then spatially normalized using an automatic nonlinear stereotaxic normalization procedure (final voxel size: 3 mm × 3 mm × 3 mm) and spatially smoothed with a three-dimensional Gaussian filter (6 mm fullwidth-half-maximum). The template image for spatial normalization was based on the average data provided by the Montreal Neurological Institute (Mazziotta et al. 1995) and conforms to a standard coordinate referencing system (Talairach and Tournoux 1988).

The images were analyzed using a standard random-effects procedure. The time series of the functional MR images obtained from each participant was analyzed separately. The effects of the experimental paradigm were estimated on a voxel-by-voxel basis, according to the general linear model extended to allow the analysis of the fMRI data as a time series. The model included a temporal high-pass filter to remove low-frequency confounds with a period above 128 s. Serial correlations in the fMRI time series were estimated with a restricted maximum likelihood (ReML) algorithm using an autoregressive AR(1) model during parameter estimation, which assumes the same correlation structure for each voxel within each scan. The ReML estimates were then used to whiten the data.

We modeled evoked fMRI responses as boxcar functions that spanned the time interval from the beginning of a trial to the presentation of the stimulus (2250 ms), which represents an ideally constant and sustained neural activity level for the whole time interval. Boxcar functions were then convolved with a canonical hemodynamic response function, which was chosen to represent the relationship between neuronal activation and blood flow changes (Boynton et al. 1996; Friston et al. 1998). Separate regressors were included for each combination of trial type (go, no-go, relax) and session (pre- and post-treatment), which yielded parameter estimates for the average hemodynamic response evoked by each type. Go trials with response omissions and no-go trials with false alarms were modeled by separate regressors and subsequently excluded from further analyses.

Parameter estimated images from each participant and condition entered a group analysis where subjects were treated as a random effect. Here we looked at brain regions more implicated in at least one experimental condition (go and no-go) as compared to the control condition (relax trials), independently of session (pre- and post-treatment) and group (control and experimental). The resulting map of the F statistic was corrected for multiple comparisons at the cluster level (p < 0.05) through a topological false discovery rate (FDR) procedure based on random field theory (Chumbley et al. 2010), after defining clusters of adjacent vertices surviving at least an uncorrected voxel-level threshold of p < 0.001.

For each participant and region, we computed a regional estimate of the amplitude of the hemodynamic response in each experimental condition by entering a spatial average (across all voxels in the region) of the pre-processed time series into the individual GLMs. Finally, for each region, we compared the two groups (control and experimental) with respect to all possible combinations of session and condition levels (pre-treatment/go, pre-treatment/no-go, post-treatment/go, post-treatment/no-go) by submitting the regional hemodynamic responses to a Mann–Whitney test. In both fMRI and behavioral analyses, we did not apply a parametric test due to the small sample size. Indeed, when the sample size is small, normality testing methods are less sensitive about non‐normality and there is chance to detect normality despite having non‐normal data. For this reason, we followed the general recommendation to use a non-parametric instead of a parametric test (Mishra et al. 2019). For this and the following analyses, a Bonferroni correction was applied to account for multiple comparisons (*p* = 0.05/N = 4, number of combinations of session and condition levels, see above).

For a qualitative comparison, we also used previous collected data on healthy control subjects (Di Russo et al. 2016). As already described in the original paper, separate regressors were included for each trial type (go, no-go, relax), which yielded parameter estimates for the average hemodynamic response evoked by each type. This allowed us to obtain a statistical parametrical map (voxel-level, 0.001 unc; cluster level, 0.05 FDR-corrected) showing brain regions more implicated in at least one experimental condition (go and no-go) as compared to the control condition (relax trials) in a representative healthy subject (see Fig. 4C). For each healthy subject, we computed a regional estimate of the amplitude of the hemodynamic response in area iFg by entering a spatial average (across all voxels in the region) of the pre-processed time series into the individual GLMs. A normative value for the iFg activity was thus obtained by averaging these estimates for each experimental condition.

### Behavioral Analysis

Behavioral performance during the DRT was assessed by measuring response time (RT) for correct trials in the Go condition and accuracy measured as omission error percentage (OM%, i.e., missed responses to targets in the Go condition) and commission error percentage (CE%, i.e., responses to non-targets in the No-Go condition). We compared the two groups (control and experimental) with respect to pre- and post-treatment sessions by submitting these measures to a Mann–Whitney test.

## Results

### Behavior

The Mann–Whitney test showed no significant differences between control and experimental groups in the behavioral performance in either pre- or post-treatment sessions. Results are detailed in Table 2. Only trials associated with correct responses were included in all the subsequent analyses. However, it should be noted that the behavioral data collected on the same patients outside the scanner revealed significant benefits in terms of processing speed and response accuracy (reduction of omission and commission errors) during the post-treatment session, but only in the experimental group (see Berchicci et al. 2019 for more details).

**Table 2:** Behavioral analysis on response time and accuracy: Descriptive parameters and statistical results of the Mann–Whitney test comparing the two groups of patients

Behaviour	Session	Mean SD		Mean rank		Statistics	
		Experimental group	Control group	Experimental group	Control group	Mann Whitney	p value
RT (ms)	Pre	921 ± 178	823 ± 214	5.00	3.67	5.00	0.57
	Post	877 ± 56	793 ± 143	5.20	3.33	4.00	0.39
OM (%)	Pre	25.6 ± 19.2	16.2 ± 10.4	5.20	3.33	4.00	0.39
	Post	6.7 ± 6.5	10.6 ± 8.9	4.00	5.33	5.00	0.57
CE (%)	Pre	20.8 ± 16.6	11.11 ± 8.7	5.20	3.33	4.00	0.39
	Post	21.4 ± 16.8	3.7 ± 5.23	5.80	2.33	1.00	0.07

*RT* reaction time, *OM* omission, *CE* commission error

### fMRI

**Fig. 2:**
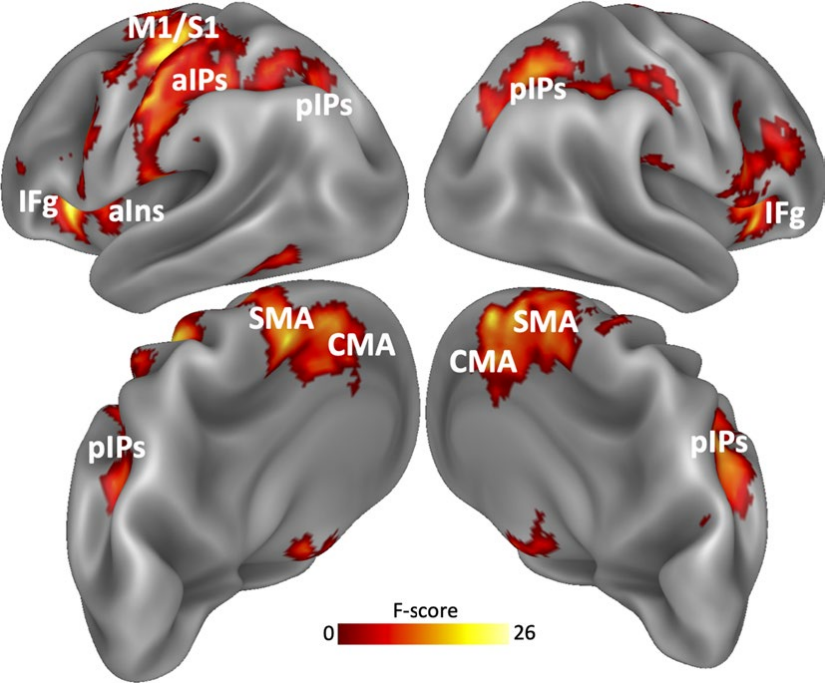
Whole-brain activation map. Regions activated by the omnibus F-contrast comparing go and no-go with relax trials, independently of session and group. Activations are rendered on reconstructions of the lateral and mesial/posterior surfaces (top and bottom panels, respectively) of the two cerebral hemispheres of the Conte69 atlas (Van Essen 2005). Labels as follows: *M1/S1* primary motor/somatosensory cortex, *aIPs* anterior intraparietal sulcus, *pIPs* posterior intraparietal sulcus, *aIns* anterior insula, *iFg* inferior frontal gyrus, *SMA* supplementary motor area, *CMA* cingulate motor area

Figure 2 shows an “omnibus” F-contrast (any condition > relax) revealing the involvement of a distributed network including the hand territory of the primary motor and somatosensory areas (M1 and S1), the anterior intraparietal sulcus (aIPs) and in the anterior insula (aIns) of the left hemisphere (contralateral to the responding hand). Strong activations were also bilaterally found in the supplementary and cingulate motor areas (SMA and CMA), posterior intraparietal sulcus (pIPs) and in the pars opercularis of the inferior frontal gyrus (iFg). The anatomical location of local maxima in each of these brain regions is shown in Table 3. A similar network of regions was observed in healthy subjects (Di Russo et al. 2016; Sulpizio et al. 2017).

**Table 3:** MNI coordinates of the local maxima of the brain regions activated during the omnibus F-contrast

MNI coordinates				
Region	Hemisphere	X	Y	Z
M1/S1	LH	− 30	− 31	55
aIPs	LH	− 48	− 19	52
pIPs	LH	− 24	− 64	43
pIPs	RH	27	− 58	46
Ins	LH	− 30	2	4
iFg	LH	− 30	23	4
iFg	RH	30	23	− 2
SMA	LH	− 3	− 7	55
SMA	RH	12	2	55
CMA	LH	− 6	14	49
CMA	RH	6	29	46

Labels as in Fig. 2

*LH* left hemisphere, *RH* right hemisphere

**Fig. 3:**
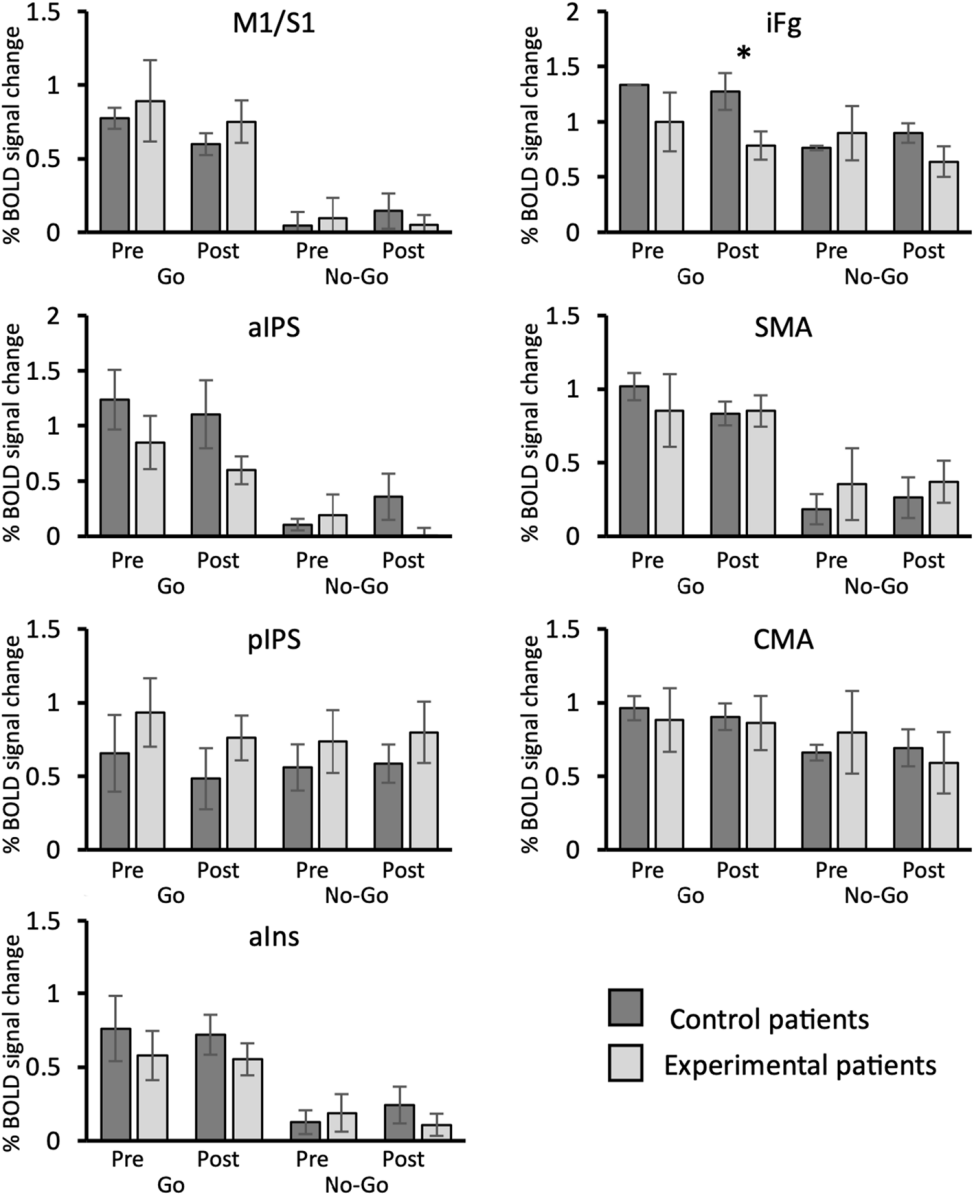
fMRI regional analysis. Plots show the percent BOLD signal change as a function of group (experimental and control), session (pre and post) and condition (go and no-go). *p = 0.01

We thus submitted the BOLD signal change estimated in each region activated by the “Omnibus” whole-brain contrast to a Mann–Whitney test to verify any group-related differences on brain activity as a function of both experimental condition (go and no-go) and session (pre- and post-treatment). These statistical results are detailed in Table 4 and in Fig. 3. The Mann–Whitney test showed significant differences between control and experimental groups only in the iFg. Interestingly, such a difference was observed only in the post-treatment session and only for the go trials. Specifically, the experimental group exhibited a significant lower iFg activity as compared to the control group (U = 7.00; p = 0.01; *η*_*p*_^2^ = 0.42).

**Table 4:** fMRI Regional analysis on BOLD signal change: Descriptive parameters and statistical results of the Mann–Whitney test comparing the two groups of patients

Region	Session	Condition	Mean ± SD		Mean rank		Statistics	
			Experimental group	Control group	Experimental group	Control group	Mann Whitney	p value
M1/S1	Pre	Go	0.89 ± 0.62	0.77 ± 0.12	4.40	4.67	7.00	1.00
		No-go	0.10 ± 0.32	0.05 ± 0.13	4.60	4.33	7.00	1.00
	Post	Go	0.75 ± 0.31	0.60 ± 0.16	5.00	3.67	5.00	0.57
		No-go	0.05 ± 0.15	0.14 ± 0.21	4.00	5.33	5.00	0.57
aIPs	Pre	Go	0.85 ± 0.54	1.24 ± 0.47	3.80	5.67	4.00	0.39
		No-go	0.19 ± 0.28	0.11 ± 0.53	4.60	4.33	7.00	1.00
	Post	Go	0.60 ± 0.42	1.11 ± 0.09	3.80	5.67	4.00	0.39
		No-go	0.00 ± 0.16	0.36 ± 0.36	3.40	6.33	2.00	0.14
pIPs	Pre	Go	0.93 ± 0.52	0.66 ± 0.45	9.30	7.17	22.00	0.43
		No-go	0.73 ± 0.34	0.56 ± 0.36	9.10	7.50	24.00	0.56
	Post	Go	0.76 ± 0.46	0.48 ± 0.27	9.40	7.00	21.00	0.37
		No-go	0.80 ± 0.47	0.59 ± 0.23	9.60	6.67	19.00	0.26
aIns	Pre	Go	0.58 ± 0.37	0.76 ± 0.37	4.00	5.33	5.00	0.57
		No-go	0.19 ± 0.24	0.13 ± 0.24	4.60	4.33	7.00	1.00
	Post	Go	0.55 $\pm$ 0.29	0.72 ± 0.14	4.00	5.33	5.00	0.57
		No-go	0.11 ± 0.17	0.24 ± 0.22	3.80	5.67	4.00	0.39
iFg	Pre	Go	1.00 ± 0.60	1.34 ± 0.00	7.70	9.83	22.00	0.43
		No-go	0.90 $\pm$ 0.29	0.76 ± 0.29	9.10	7.50	24.00	0.56
	Post	Go	0.79 ± 0.55	1.28 ± 0.04	6.20	12.33	7.00	0.01*
		No-go	0.64 ± 0.31	0.90 ± 0.15	7.10	10.83	16.00	0.15
SMA	Pre	Go	0.85 ± 0.55	1.02 ± 0.16	7.90	9.50	24.00	0.56
		No-go	0.35 $\pm$ 0.24	0.18 ± 0.14	8.70	8.17	28.00	0.88
	Post	Go	0.85 ± 0.55	0.83 ± 0.18	8.70	8.17	28.00	0.88
		No-go	0.37 ± 0.32	0.26 ± 0.24	9.10	7.50	24.00	0.56
CMA	Pre	Go	0.88 $\pm$ 0.48	0.96 ± 0.14	8.30	8.83	28.00	0.88
		No-go	0.80 $\pm$ 0.41	0.66 ± 0.16	8.40	8.67	29.00	0.96
	Post	Go	0.86 $\pm$ 0.63	0.90 ± 0.09	7.90	9.50	24.00	0.56
		No-go	0.59 ± 0.47	0.69 ± 0.22	8.00	9.33	25.00	0.64

Labels as in Fig. 2. Significant result is marked by asterisk

Additionally, we evaluated whether this reduced activity might reflect a normalization trend. To this aim we compared this result with that observed in healthy subjects in our previous study (Di Russo et al. 2016). Although in that study we did not show the mean BOLD response of area iFg, here we computed the mean iFg response elicited by go trials in the sample of healthy subjects and used it for a qualitative comparison with the response observed in the two groups of patients. As expected, the difference found between experimental and control groups during the post-treatment session goes towards a normalization of the iFg activity, since the activity observed in the experimental group (i.e., percent BOLD signal change $\pm$ SE = 0.79 $\pm$ 0.55, see Table 4) approached to the value observed in healthy subjects (percent BOLD signal change $\pm$ SE = 0.72 $\pm$ 0.09).

**Fig. 4:**
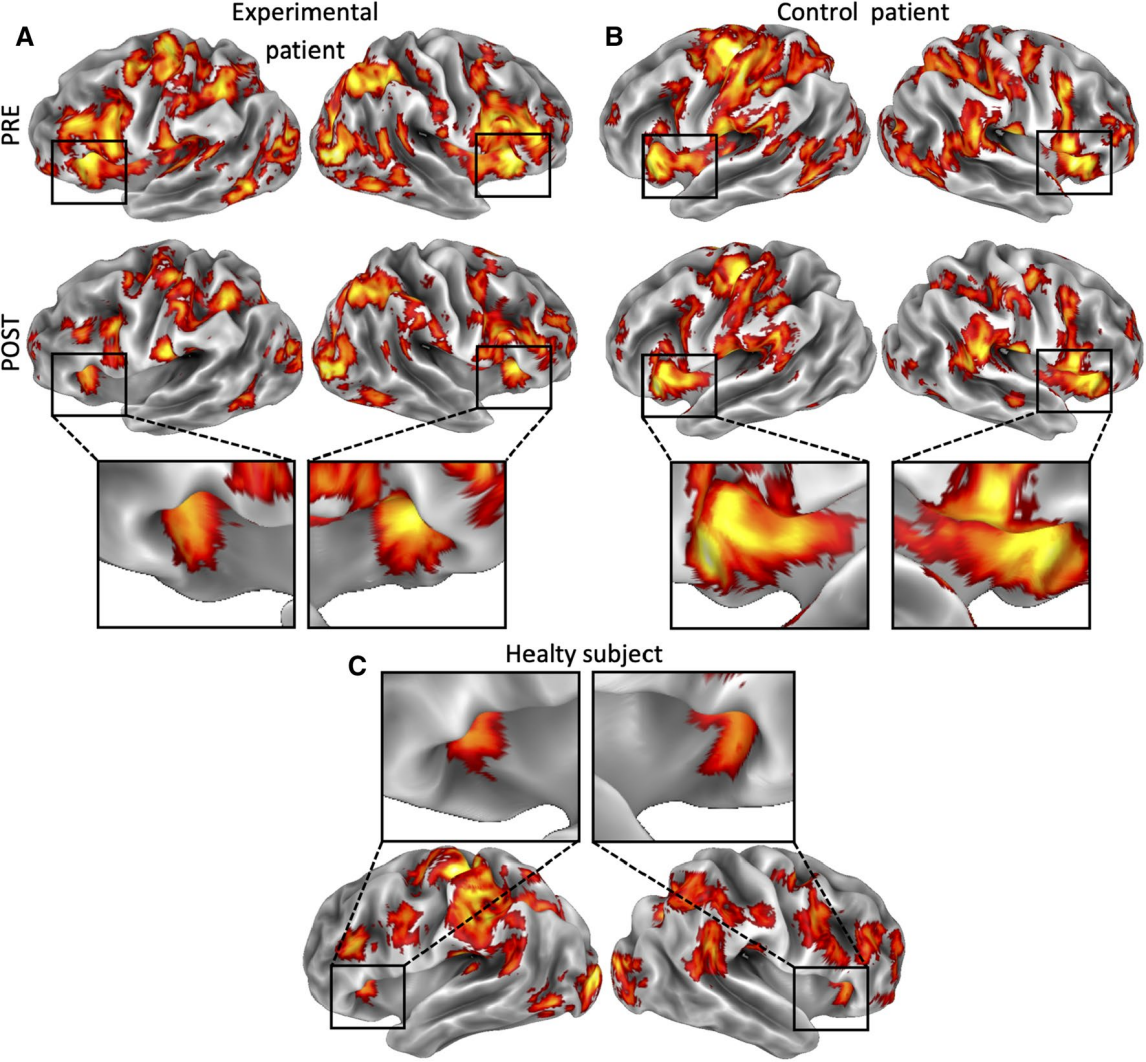
Individual activation maps for the go > relax contrast. Single subject activation maps displayed on the cortical surface reconstruction of both left and right hemispheres of a representative experimental patient (**A**), a representative control patient (**B**) during both pre- (top rows) and post-treatment (bottom rows) sessions, and a representative healthy subject from Di Russo et al. 2016 (**C**). Close-up views show the iFg activation of both patients during the post-treatment session in comparison with that of the healthy subject. All these maps were thresholded at p < 0.05 FDR-corrected at the cluster level, with a cluster-forming threshold of p < 0.001 uncorrected

Figure 4 shows single-subject activation maps for the go > relax contrast displayed on the cortical surface reconstruction of both left and right hemispheres of a representative experimental patient (Fig. 4A) and a representative control patient (Fig. 4B) during both pre- (top rows) and post-treatment (bottom rows) sessions. These maps were also compared to the go > relax map obtained in a representative healthy subject from Di Russo et al. (2016) (Fig. 4C). Inspection of these individual maps reveled large swaths of activation in both experimental and control patients, especially in the pre-treatment session (Fig. 4A, B, top rows). After HBP treatment, the experimental patient showed a remarkable reduction of the overall cortical activation, especially in correspondence of area iFg (Fig. 4A, bottom row). Close-up views show the iFg activation of both patients during the post-treatment session in comparison with that of the healthy subject (Fig. 4C). This qualitative comparison suggests the presence of a normalization of the iFg activity, but only in the experimental patient. The control patient, indeed, although exhibited a post-treatment reduction of activation in more posterior regions, did not show a remarkable reduction of iFg activation (Fig. 4B) as compared to the experimental patient (Fig. 4A). These maps confirm that the iFg hyperactivation become weaker especially after HBP treatment.

## Discussion

We tested the effect of an exoskeleton-assisted rehabilitation training over the neural correlates involved in cognitive processing of motor planning and response execution in a sample of MS patients. To our knowledge, this is the first fMRI study implementing exoskeleton in multidisciplinary rehabilitation training for MS patients measuring neural, behavioral, functional, and clinical data. Previous reports demonstrated that HBP application improves mobility in MS patients, with effects on brain activity studied by electroencephalographic measures based on event-related potentials and mainly ascribed to the prefrontal cortex (Di Russo et al. 2013; Berchicci et al. 2019). By using fMRI here we showed that HBP intervention induces a cortical activity’s reduction in a specific prefrontal region, i.e., area iFg, which is considered as critical for inhibiting response tendencies for behavioral and attentional control (Aron et al. 2004). This suggests that the HBP intervention might be useful to mitigate the cortical hyperactivity associated to MS, although a large-scale study is required to confirm this view.

In a previous ERP study on healthy young subjects (Di Russo et al. 2016) we applied the same discriminative visuo-motor task (Go/No-go task) used here to study brain localization and timing of neural activity underlying anticipatory proactive mechanisms. The main finding of such a study was that iFg is the core structure for cognitive preparation and proactive inhibition. According with this view, a series of neuroimaging and neuropsychological evidence converges in suggesting that the iFg (especially the right one, r-IFg) plays a crucial role in inhibitory processes across a range of tasks, requiring suppression of response tendencies (Aron et al. 2004, 2014). Also, a recent fMRI metanalysis of Go/NoGo and Stop Signal (SST) studies revealed that the r-IFg is mainly involved in proactive control while the right middle frontal gyrus is mainly involved in reactive control (Gavazzi et al. 2020). Notice that the present whole-brain maps on both patients and healthy controls show a bilateral iFg involvement. Thus, the observed activation of the left iFg could be an unexpected result. However, although the literature on cognitive control converges on the general idea that response inhibition is lateralized to the right hemisphere (see Simmonds et al. 2008; Aron et al. 2014; Gavazzi et al. 2020 for recent reviews and meta-analyses), there are also a number of fMRI studies on response inhibition which failed to observe a right iFg dominance (Swick et al. 2008; Di Russo et al. 2016; Sulpizio et al. 2017; Pan et al. 2021). Moreover, Gavazzi et al. (2019) described a patient with damage to almost the entire right hemisphere who exhibited spared inhibitory functions, likely mediated by the left homotopic iFg.

Taken together, previous and current results suggest that iFg is a key node of the proactive inhibition mechanism: its activity, indeed, starts before the stimulus onset and it is released concomitantly to stimulus appearance. We have also proposed that the prefrontal negative activity is responsible for a proactive response inhibition as far as the movement is not needed; basically, if the need to perform an action is approaching, but it is not yet the time to execute it, the prefrontal cortex works like a brake to freeze the action until the right time (Di Russo et al. 2016). This evidence supports the idea that area iFg is the highest stage of neural integration in the perception-action cycle, playing thus a critical role in action monitoring. Note that behavioral performance and brain activity of MS patients are markedly deteriorated during motor preparation and execution (Aminoff and Goodin 2001; Larson et al. 2002; Whelan et al. 2010). The effect of HBP we observed here in terms of a reduced iFg activity seems to reflect a compensatory mechanism, similarly to that previously observed in old healthy adults. Although prefrontal control becomes progressively stronger in this population (Berchicci et al. 2012), a physically active lifestyle appears to counteract such an over-recruitment during action preparation (Berchicci et al. 2013a).

Here we also evaluated whether such a reduction of prefrontal activity could be explained as a retour to a normal range. To this aim, we performed a qualitative comparison between the iFg activity observed after HBP treatment in the experimental group with that previously observed in healthy subjects (Di Russo et al. 2016). We found that the amount of iFg activity observed in patients undergoing the HBP treatment approached that observed in healthy subjects. The present data highlight the importance of the HBP treatment in reducing cortical hyperactivity. A widespread cortical hyperactivity has been associated with a series of neurodegenerative diseases including MS. For instance, a meta-analysis by Kollndorfer et al. (2013) revealed that MS patients exhibited higher neuronal activation (as compared to healthy subjects) in the ventrolateral prefrontal cortex (VLPC) during tasks on executive functions. It has been proposed that the VLPC, also including the pars opercularis of iFg, mediates cognitive control through a wide range of functions such as memory, motor inhibition, action updating and reflexive reorienting (i.e., reorienting attention towards unexpected events; see Corbetta et al. 2008 for a review) (see Levy and Wagner 2011 for a meta-analysis). Notably, these cognitive capacities are often impaired in MS (Benedict et al. 2006; Rao et al. 1991). However, deficits in executive functions, such as abstract and conceptual reasoning, fluency, planning, are less frequently observed in MS patients as compared to deficits in memory and processing speed (Chiaravalloti and DeLuca 2008; Benedict and Zivadinov 2011).

Overall, the cortical hyperactivity observed in MS patients might be explained by overreaching compensatory mechanisms, which have also been associated to cognitive impairments in other diseases, such as major depression (Diener et al. 2012) or Alzheimer’s disease (Browndyke et al. 2013). Present findings indicate that the use of HBP during rehabilitation intervention normalizes the prefrontal activity, mitigating the prefrontal cortical hyperactivity associated to MS. Similar to our results, a recent study, which assessed the effect of multidisciplinary rehabilitation on the brain activity of MS patients, revealed a significant reduction in the activity of brain areas related to action-related tasks (Péran et al. 2020).

Moving to the typical neurological and rehabilitation measures, we observed an improved performance in both groups after the treatments in several functional and neurological tests. This is of great importance, because it means that rehabilitation processes did properly work in mitigating the MS symptoms. However, the HBP was more effective than the traditional rehabilitation protocol in reducing the fatigue perception (FSS). Indeed, only the experimental group performance on this scale was much better than before. The FSS is a fatigue severity scale designed to assess disabling fatigue. It is clinically relevant as fatigue is a prominent disabling symptom in a variety of medical and neurologic disorders, including MS. Notably, in previous studies, we have already showed a correlation between prefrontal activities and fatigue perception (Berchicci et al. 2013b; Menotti et al. 2014). Also, in this study, we observed a reduced prefrontal activity together with a reduction of self-report fatigue, in line with previous interpretation on conscious perception of fatigue. Further, since the HBP should exert its effect throughout proprioceptive mechanisms, it might be responsible for fatigue perception modification. Besides the interpretation of this finding, which needs to be further supported, the decreased perception of fatigue is very important, because it is one of the most debilitation symptoms in MS.

A final note goes to the behavioral results. Although we observed that the use of the HBP induces changes in the activation of the prefrontal cortex, we did not find any behavioral improvement after the experimental treatment. This weakens the interpretation of a potential compensatory mechanism induced by the HBP treatment. However, the lack of significant group differences in the behavioral performance might be explained by the small amount and variability of data. Indeed, data collected on the same sample of patients in a previous ERP study of ours (Berchicci et al. 2019), by using the same Go/NoGo task before and after rehabilitation intervention, revealed a significant RT reduction and increase of accuracy from pre- to post-treatment, but only in the experimental group. A possible explanation for the different behavioral effects obtained on the same sample between the previous and the present study regards the number of trials (eg., Di Russo et al. 2016; Sulpizio et al. 2017; Berchicci et al. 2020). Indeed, each patient responded to 320 go trials during the ERP experiment and to 72 go trials during the present fMRI experiment. The low number of trials in fMRI may have contributed to keep standard deviation high, reducing also the likelihood to observe any significant difference in the behavioral performance. Although more data are needed to bring out the behavioral advantage also with the fMRI sampling, based on Berchicci et al. (2019) we can conclude that the experimental treatment induced benefits in terms of processing speed and response accuracy.

There are several limitations in the present study which could be addressed in future work. The first limitation is the low number of patients recruited. The present results come from a small sample of patients that might have a specific range of mobility deficits, not necessarily generalizable to the general MS population. Future studies, using larger sample of MS patients, are needed to corroborate present results. In particular, these large-scale studies should benefit in using parametric analyses, such as factorial ANOVAs, in order to reveal significant interactions between group and treatment variables. Another possible limitation is the indirect comparison of brain activation of MS patients with the data of young healthy controls enrolled in our previous study in which the same task was used (Di Russo et al. 2016). So, the results obtained in the present pilot study need to be furtherly verified by a formal direct comparison between patients and healthy subjects. To this aim, future studies should enroll healthy participants that are gender-, education- and age-matched to MS patients, and submit them to the same experimental protocol employed for MS patients.

In conclusion, the present preliminary results reveal that the HBP intervention could be effective in mitigating the MS symptoms and inducing changes at the brain level. In particular, the HBP reduces the fatigue perception likely reducing cognitive processing in prefrontal cortex. This suggests that walking with an exoskeleton may help enable people with multiple sclerosis to walk more efficiently by reducing the energy and muscle activity needed to walk. Compared to a robotic exoskeleton, the HBP system has several advantages (it is cheaper, lighter and compact, e.g., portable in a case) that permit patients to stay at home during the HBP treatment, allowing large savings in welfare costs. Although the HBP is not a cure for MS, this exoskeleton may be a valid support to the standard treatment of MS.

## Data Availability

Present data will be made available on request in compliance with the requirements of the funding institutes, and with the institutional ethics approval.
